# The effect of screw thread length on initial stability of Schatzker type 1 tibial plateau fracture fixation: a biomechanical study

**DOI:** 10.1186/s13018-016-0484-9

**Published:** 2016-11-22

**Authors:** Ahmet Salduz, Fevzi Birisik, Gokhan Polat, Bugra Bekler, Ergun Bozdag, Onder Kilicoglu

**Affiliations:** 1 Department of Orthopedic and Traumatology, Istanbul Faculty of Medicine, Istanbul University, 34093 Istanbul, Turkey; 2 Laboratory of Biomechanics and Strength of Materials, Faculty of Mechanical Engineering, Istanbul Technical University, Istanbul, Turkey

**Keywords:** Tibial plateau fractures, Cancellous screw, Thread length, Cyclic loading, Biomechanical test

## Abstract

**Background:**

This study compares the cyclic loading properties and failure loads of two screw combinations on a synthetic Schatzker type 1 tibia fracture model. Our hypothesis was that after adequate compression with first a partially threaded screw, addition of a fully threaded screw would provide more stability than an addition of a second partially threaded screw.

**Methods:**

The Schatzker type 1 tibial plateau fracture model was created. Fixation was obtained in group A (*n* = 10) with two partially threaded screws and in group B (*n* = 10) with one fully threaded screw and one partially threaded screw. Load-displacement evaluation was made at each 1000-cycle interval up to 10,000 cycles. Failure load was identified as the load creating a 2-mm displacement. Two-factor (groups and periods) repeated measurement analysis of variance and independent sample *t* tests were used.

**Results:**

According to the two-factor repeated analysis, there was no significant difference for periods (*p* = 0.29) and time-period interaction (*p* = 0.59) (Wilk’s Lambda *F* value, 1.507 and 0.871, respectively). In the test of between-subject effects, there was no significant difference between groups in terms of cyclic loadings (*p* = 0.06, *F* = 4.065). However, in the *t* test for each 1000-cycle interval, the value of mean displacement in group B was significantly lower than that in group A in the initial, 1000-, 2000-, and 3000-cycle intervals (*p* = 0.023, 0.031, 0.025, 0.043, respectively). The mean displacement and standard deviations increased with the number of cycles. The mean range of displacement initially was 0.66 mm for group A and 0.36 mm for group B. The mean range of displacement after 10,000 cycles was 0.79 mm for group A and 0.44 mm for group B. The mean failure load value was 682 ± 234 N for group A and 835 ± 245 N for group B. In independent sample *t* tests, there were no significant differences between the two groups in terms of failure load (*p* > 0.05).

**Conclusions:**

Obtaining fixation with one partially and one fully threaded screw can minimize displacement at the fracture site at early cyclic loadings.

## Background

The treatment goal for tibial plateau fractures is anatomic reduction and rigid fixation to allow early mobilization. Several fixation methods have been described for rigid osteosynthesis. Percutaneous fixation using a partially threaded cannulated screw is one of the methods, which is reported by some authors with a high rate of success [1–3]. In this technique, generally, partially threaded screws are used and it is recommended that the threaded part of these screws remain distal to the fracture line to obtain compression on the fracture line. The characteristics of screws such as the thread length, diameter, thread pitch, and length of the screws affect biomechanical properties of the fixation [4–12]. This biomechanical strength is important to start early mobilization. Mathur et al. reported that some patients had loss of knee range of motion because of delayed knee mobilization due to the lack of the rigid fixation [13]. One reason for lack of rigid fixation may be loss of compression at the fracture site. If the compression at the fracture site is lost, motion between fracture fragments may occur, which may result in non-union or malunion. To our knowledge, there is no study in the literature comparing fixation strengths for different screw combinations. Our hypothesis is that different screw combinations may have different fixation strengths and specifically, addition of a fully threaded screw after obtaining fracture compression using a partially threaded cannulated screw may increase the rigidity of fixation.

## Methods

The cyclic loading properties and failure loads of screw combinations with different thread lengths were compared biomechanically on a Schatzker type 1 tibia fracture model using synthetic bone.

Twenty identical synthetic right tibia bones representing healthy bones without osteoporosis (Synbone, Type: 1149, Synbone AG, Swiss) were used in the study. According to the manufacturer, although their synthetic tibia model is not as strong as the natural bone, it had very favorable feedback from biomechanical testing facilities and it was demonstrated with ballistic testing that it fractures in a manner very similar to the live bone [14] (length 387 mm; tibial plateau width 74 mm; shaft diameter 27 mm; canal diameter 9.5 mm; material: cortical/hard cancellous bone). We preferred cancellous fixation screws considering the fact that the bone structure of the proximal tibia is mostly cancellous. These 20 bones were divided into two groups of 10 and labeled as group A and group B.

**Fig. 1:**
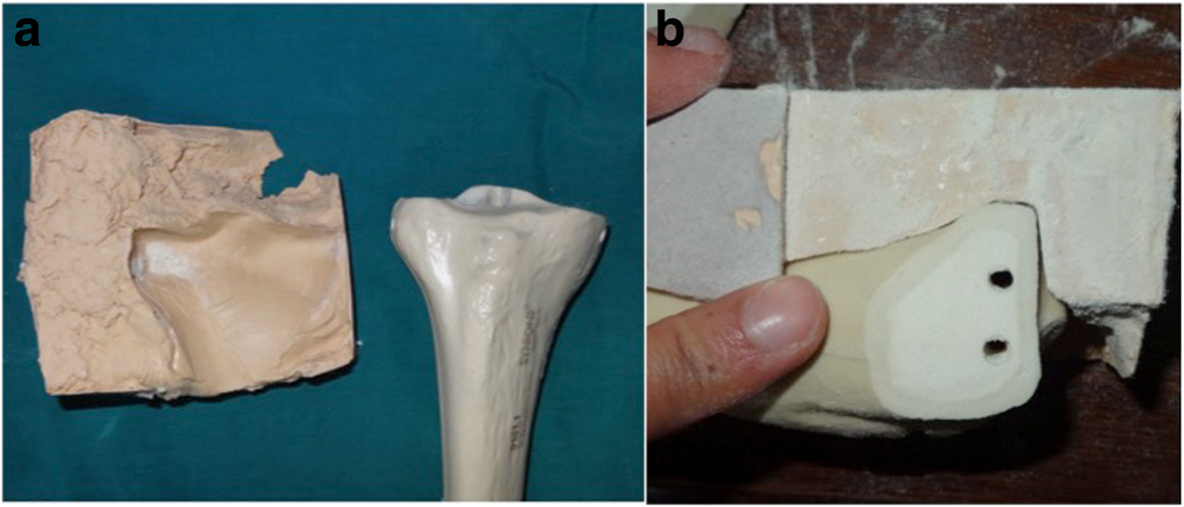
**a** Synbone tibia model with the putty template. **b** The same model after the holes have been drilled and the osteotomy was made

In order to get standardized results, we needed to standardize screw placement and osteotomy sites. To standardize screw drill holes, first, we drilled two holes in a tibia from lateral to medial with 4.5-mm drill bits, as we would normally do in routine surgeries of Schatzker type 1 tibial plateau fractures. Then, without removing the drills from the bone, we placed them in a durable card box and poured the putty so as to completely encase the bone and the drills. As the polyester steel putty hardened it took the shape of the materials. Then, we pulled out the drills from the bone. Because the putty also hardened around the drills, the location, size, and direction of the drill holes could now be replicated with exact accuracy. This way, when we put a new tibia in the hardened putty template, drills would go through the tunnel inside the putty and we could target them into the bone in a standardized manner (Fig. 1a).

To create an osteotomy template, we first created a template of the proximal tibia by using the same method of encasing the bone about halfway inside the putty and letting it harden around the bone. We decided to create an osteotomy line 1.5 cm from the lateral edge of the tibial plateau. Then, we sliced the template in a way so that when the bones would be later placed on it, part of the tibia to be osteotomized would hang at the side of the template (Fig. 1b). In this way, a standardized manner of creating Schatzker type 1 fractures (Orthopedic Trauma Association (OTA) classification: 41-B1) and drills holes was established.

**Fig. 2:**
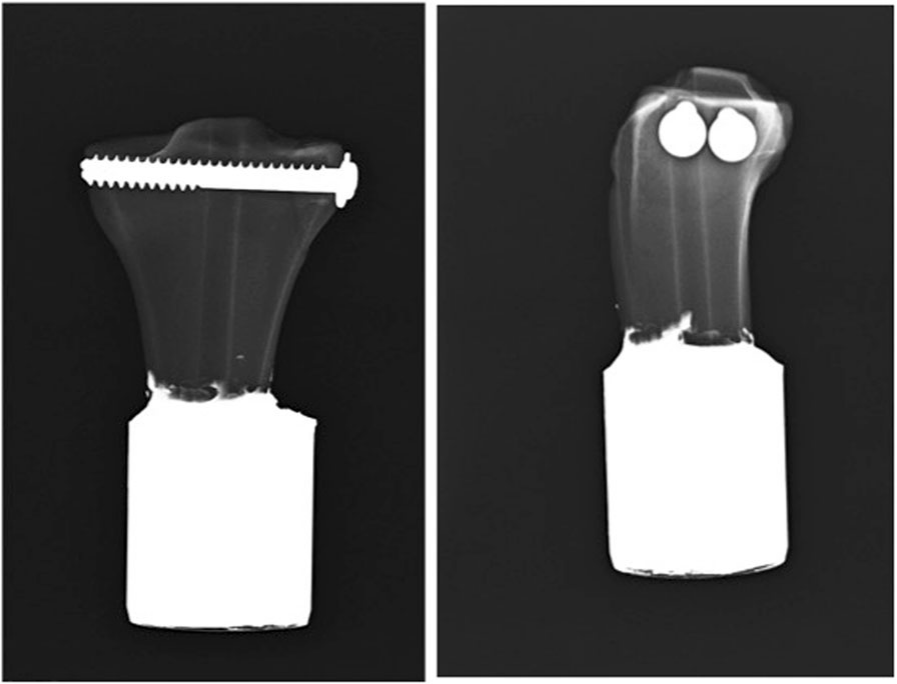
AP and lateral X-rays of the bone model after screw fixation

After a model was osteotomized, the fragment was repositioned and fixed with combinations of two group screws. All screws were 75 mm in length and 6.5 mm in diameter. Two partially threaded cancellous screws were used for group A and one partially threaded screw and one fully threaded screw was for group B (Tasarımmed®, Istanbul, Turkey). The partially threaded screws in group B were always placed in the posterior hole, and the fully threaded screws were always placed in the anterior hole. Before the screws were placed, the screw path was tapped and they were tightened with screwdrivers at a torque limit of 1.5 N. This limit was chosen to make sure that all the screws were tightened with the same amount, and the screwdriver with 1.5-N limits is used routinely in our daily practice. Tibia specimens were cut perpendicularly to the long axis of the bone at 13 cm of the distance from the proximal end. Each sample was embedded in a plastic cylinder, which was 5 cm in diameter and 5 cm in length, using polyester steel putty. This was done so that the distal end of the tibia would be suitable for adapting on a testing machine. X-rays of the specimen are shown in Fig. 2.

### Biomechanical testing

**Fig. 3:**
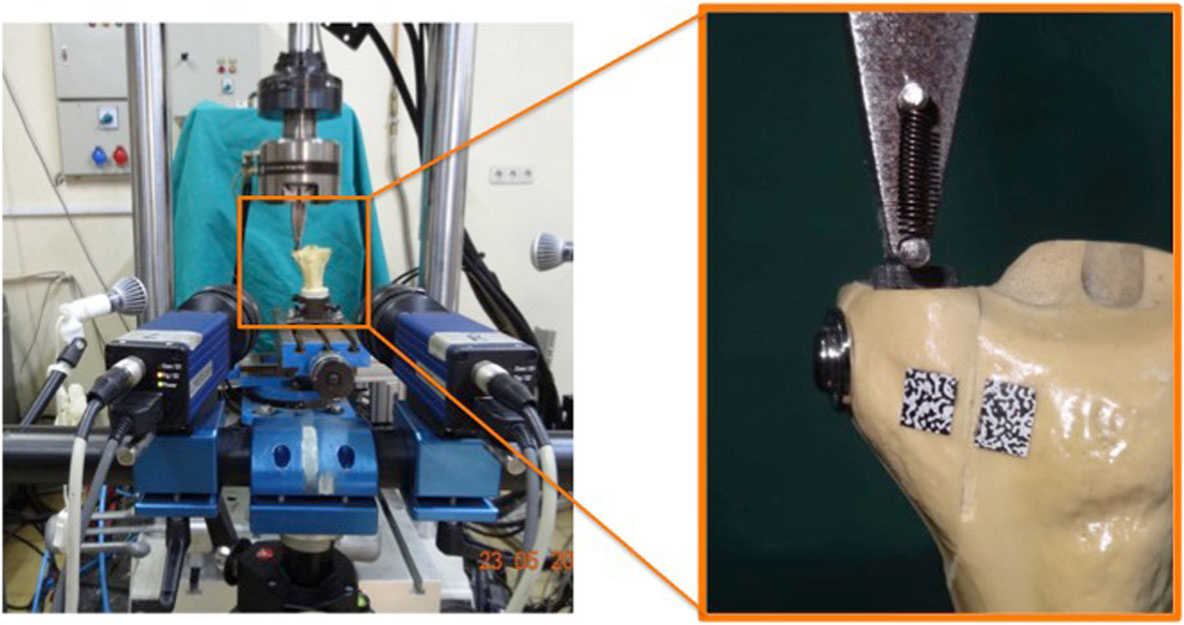
Experiment setup with two cameras pointed towards the bone model. The figure on the *right* is the close-up of the model

The material test device (MTS 858 Mini Bionix™ II) was set up in such a way as to have direct loading on the fracture fragment (Fig. 3). This simulated the action shearing forces on the tibial plateau in full knee extension. We chose this position because it creates the greatest shearing forces on the tibiofemoral joint [15]. A rectangular-shaped piece of rubber was fixed on the fragment of the bone to distribute the load on a greater joint surface. Grid stickers were pasted on both sides of the fracture line on the anterior surface of the tibia. The stickers were registered digitally, and their movements were analyzed using two high-tech cameras that were able to do a 3D analysis (Vic3D 3D Digital Imaging Correlation (DIC), Correlated Solutions Inc.), although we only had a coronal plan analysis of displacement. We defined displacement as 2 mm of movement along any axis in the space on the basis that intra-articular fractures are usually treated operatively if there is more than 2 mm of articular step off. Pictures were taken as four frames per second for each 1000-cycle interval and load to failure test.

#### Cyclic loading (dynamic loading)

A load to failure test was performed as a preliminary test on a sample from each group to identify the load to be used in cyclic loading tests. The lowest failure load among samples was selected, and two thirds of this value, 300 N, was used as the upper limit in the cyclic loading tests. For the cyclic loading, frequency and preload were determined as 5 Hz and 10 N, respectively. Between each 1000-cycle interval, the image analysis software recorded a load-displacement evaluation under 300 N of load. This load was not applied instantly but incrementally in 30 s with an increase of 10 N/s to detect movement with better accuracy. After 10 cyclic loading intervals, failure load values were obtained by continuous loading. The number of cycles was determined as 10,000 cycles according to similar studies in the literature [7, 16, 17].

#### Load to failure (static loading)

**Fig. 4:**
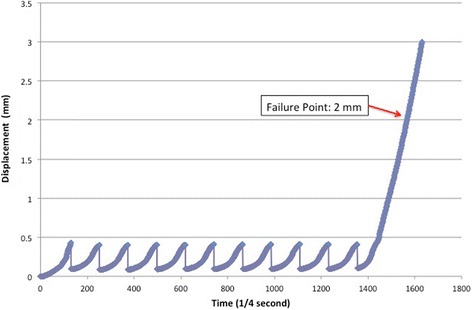
Time-displacement graphic of one sample. Initial and 10 cyclic loading tests were seen as 11 peaks total. Displacement was increasing because of the load to failure test

Following the cyclic loading, a load to failure test was performed. The loading speed was identified as 5 mm/min, and the loading was maintained until 3 mm of displacement. The displacements were detected with an optical camera during this loading phase. The load that caused a 2-mm displacement was identified as the failure load. In one of the load to failure tests, technical errors occurred and meaningful values were not obtained. This measurement was excluded from the study. An example load-displacement graphic, consisting of an initial and 10 cyclic intervals and load to failure tests, is demonstrated in Fig. 4.

### Statistical assessment

Independent sample *t* tests were used to analyze each period and failure load. Two-factor (groups and periods) repeated measurement analysis of variance was used to simultaneously analyze all cyclic loadings. A *p* value <0.05 was considered as significant. All statistical analyses were performed using SPSS software (version 20.0. Armonk, NY: IBM Corp.) on a personal computer.

## Results

**Fig. 5:**
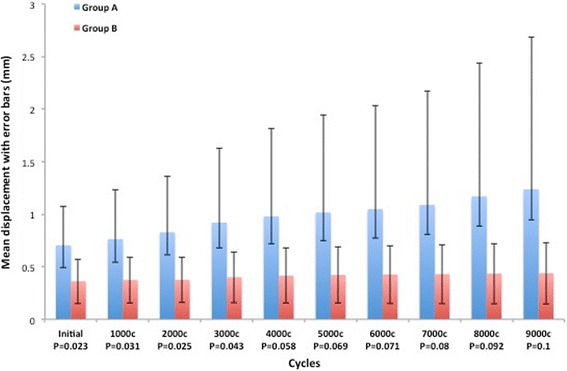
The graph shows the differences between groups A and B. Up to 3000 cycles, group B has significantly lower displacement values than group A

The first statistical analysis that we performed concerned mean displacement values of two groups at specific cycles to assess any differences in stability of the fixation of the fracture site. When each period was evaluated separately with an independent *t* test, the value of mean displacement in group B was significantly lower than that in group A in the initial, 1000-, 2000-, and 3000-cycle intervals (*p* = 0.023, 0.031, 0.025, 0.043, respectively). The mean range of displacement initially was 0.66 mm for group A and 0.36 mm for group B. The mean range of displacement after 10,000 cycles was 0.79 mm for group A and 0.44 mm for group B. The mean displacement and standard deviations increased with the number of cycles (Fig. 5). After 4000 intervals, the differences between mean displacements were not statistically significant anymore.

The second statistical analysis concerned differences of mean failure loads to assess the strength of the fixation. The mean failure load value was 682 ± 234 N for group A and 835 ± 245 N for group B. There were no significant differences between the two groups in terms of failure load in independent sample *t* tests (*p* > 0.05). The mean and *p* values are shown in Table 1.

**Table 1:** The mean displacement amount at every 1000-cycle loading phase and the mean failure load values at 2-mm displacement with standard deviations

Number of cycles	Group A (mm)	Group B (mm)	*p* value
0	0.66 ± 0.37	0.36 ± 0.21	0.023
1000	0.71 ± 0.48	0.37 ± 0.22	0.031
2000	0.73 ± 0.49	0.37 ± 0.21	0.025
3000	0.74 ± 0.50	0.40 ± 0.24	0.043
4000	0.75 ± 0.51	0.41 ± 0.26	0.058
5000	0.75 ± 0.52	0.42 ± 0.27	0.069
6000	0.76 ± 0.53	0.43 ± 0.28	0.071
7000	0.77 ± 0.53	0.43 ± 0.28	0.08
8000	0.78 ± 0.54	0.43 ± 0.29	0.092
9000	0.79 ± 0.56	0.44 ± 0.29	0.1
10,000	0.79 ± 0.56	0.44 ± 0.29	0.1
Failure load (*N*)	682.00 ± 234.90	835.70 ± 245.25	>0.05

Finally, repeated measurement analysis was performed to make a comprehensive evaluation of our data, which included all cycles. This analysis was made using two factors. Of these two, the within-subjects factor was periods of each 1000 cycle and the between-subjects factor was groups. According to the multivariate tests, there was no significant difference for periods (*p* = 0.29) and time-period interaction (*p* = 0.59) (*F* value of Wilk’s Lambda test, 1.507 and 0.871, respectively). In the test of the between-subject effects, there was no statistical difference between the groups as far as how they effect displacement by themselves (*p* = 0.06, *F* = 4.065).

## Discussion

The tibiofemoral joint is subject to different types of forces in different daily activities. The reason of the failure of plateau fractures of the tibia is primarily shearing forces, which cause the fracture fragment to displace, resulting in failure of the osteosynthesis. Therefore, we preferred to test our hypothesis under conditions that create shearing forces on the tibiofemoral joint. The shearing forces are different in various daily activities. They also change with the degree of flexion and extension in the knee joint. The shearing forces are greatest at the last few degrees of extension [15, 18]. This, however, must be considered with the fact that although shearing forces are greater in extension, there is more contact area to distribute the forces so that the forces applied on unit surfaces may actually be less. According to some studies, walking creates shearing forces in the range of 0.6 × body weight, which means that shearing forces exerted on the tibiofemoral joint of an 80-kg male would be around 480 N [15, 18].

Cyclic loading test is very important for representing physiological loadings [7, 19]. We used it to simulate the partial-weight-bearing condition at the early postoperative times. Although late cyclic results are not significantly different in the two groups, our results show that fixation in group B is more durable in early cycles for the same shearing force than that in group A. This may be used to the patients’ advantage since in early rehabilitation, patients would benefit from the extra amount of secure fixation provided by the screw combination in group B.

The lag technique is still a recognized and accepted technique for optimal primary healing. According to this technique, all threads must cross the osteotomy or fracture site to generate compression. We could not find a paper analyzing different thread configurations at the fracture line in terms of biomechanical features. Our results indicate that after adequate compression with first a partially threaded screw, using a fully thread screw may increase the initial stability of fracture fixation. The loss of the significant differences after 3000 cycles can be explained by the loosening of the screws after cyclic loading for both groups.

While no studies on plateau fractures comparing screw combinations exist, there are some similar studies in the literature, although they do not compare fully and partially threaded screws [20]. Fixation of medial malleoli fractures with fully threaded cortical screw has an advantage compared to unicortical partially threaded lag screws [21, 22]. On the other hand, there was no significant difference between long and short thread screws for the fixation of the intra-capsular hip fractures by Parker and Ali [23]. Other properties of screws which effect holding power are also investigated in the literature [4, 5, 7, 8, 10, 11, 24–26].

The failure load-displacement amount was chosen as 2 mm because 2 mm is a common threshold for treating intra-articular fractures operatively. Furthermore, the number of cycles in this study can be considered low, especially compared to the standard steps/day values required for non-sedentary lifestyle in healthy adults [27]. For a normal adult, 10,000 cycles represent less than a week of walking. Nevertheless, 10,000 cycles were enough in our case for failure, which was the endpoint to our experiment. Assuming a person applies bending force 250 times a day for 6 weeks will result in almost 10.000 cycles of this motion. If a healing period of 6 weeks is assumed, the 10,000 cycles is a good estimation for a condition through healing. Additionally, 10,000 cycles was used in the biomechanical studies about fracture fixation in the literature [7, 16, 17].

Due to its experimental nature, there are several limitations to our study. The first and foremost limitations of this study were the low number of samples and the use of synthetic bone instead of real bone. Another limitation was that we chose to stick our grids right across the midpoint of the fracture line. However, the screws across the fracture line can act as a center of rotation under a lateral shearing force, causing the proximal and distal parts of the fracture to displace differently. Therefore, this study treats the fracture as a single rigid body that shows constant displacement at different corresponding points, which is a simplification of the real fracture mechanics. However, we believe that, although a limiting factor, the difference can be negligible for this sample size.

In real life, the force applied by the body weight on the tibia changes with each degree of flexion and extension and the force applied on the joint surface is different at each point along the gait cycle. However, due to practical limitations, our setup was prepared in such a way as to be an approximation by accepting the load to be constant along the cycle. Furthermore, in real life, the load applied on the joint surface is not the same at every point. However, we could not factor that in our experiment because the cyclic load was applied not by a distal femur as in real life but through a sheet-like hard rubber, which had a smaller surface area than the joint surface. Therefore, the load was concentrated on a smaller area. Since we intend to imitate a real-life biomechanical process, this is a major limitation of our study. The sheet-like rectangular rubber used to distribute the force applied by the machine on the tibia models had a hard consistency to transmit the force without much compression. Still, some amount of the force applied may be lost in compressing the rubber. Insignificant as it may be, it can be considered as a systematic error in our study. In the light of these limitations, we believe further studies with a large number of samples—preferentially real bone—are needed.

## Conclusions

Obtaining fixation with one partially and one fully threaded screw can minimize displacement at the fracture site at early cyclic loadings. This combination might be useful for the Schatzker type 1 tibial plateau fracture during the early rehabilitation period. Although we have failed to demonstrate significant differences at later cycles and failure loads, we have successfully demonstrated that different screw combinations may have different biomechanical properties at the fracture site. With further research, this fact may be translated into meaningful clinical results by adapting specific screw combinations depending on the type of fracture to achieve more secure fixation.

## Abbreviation

OTA: Orthopedic Trauma Association
